# Anti-Inflammatory Effect of Wasp Venom in BV-2 Microglial Cells in Comparison with Bee Venom

**DOI:** 10.3390/insects12040297

**Published:** 2021-03-29

**Authors:** Hyun Seok Yun, Jisun Oh, Ji Sun Lim, Hyo Jung Kim, Jong-Sang Kim

**Affiliations:** 1 School of Food Science and Biotechnology, Kyungpook National University, Daegu 41566, Korea; solideo0116@naver.com; 2 Institute of Agriculture Science and Technology, Kyungpook National University, Daegu 41566, Korea; j.oh@knu.ac.kr (J.O.); lzsunny@daum.net (J.S.L.); 3 National Institute for Korean Medicine Development, Gyeongsan 38540, Korea; indersee31@nikom.or.kr

**Keywords:** *Vespa velutina*, wasp venom, microglia, neuroinflammation, NF-κB

## Abstract

**Simple Summary:**

As the population of the yellow-legged hornet (*Vespa velutina*) spreads, this study investigated ways to utilize this resource of abundant invasive wasp species. Hymenoptera venoms, including bee venom and wasp venom, have therapeutic potential. Although the venoms are toxic to humans, the elucidation of their composition and working mechanisms has led to discoveries about their potential applications in treatment modalities for a variety of disorders. Therefore, we examined the anti-inflammatory effect of wasp venom from *V. velutina* in comparison with that of bee venom from honey bee on BV-2 murine microglial cells. Treatment with wasp venom reduced the secretion of nitric oxide and pro-inflammatory cytokines, including interleukin-6 and tumor necrosis factor alpha, from BV-2 cells activated by lipopolysaccharide (LPS). Western blot analysis revealed that wasp venom and bee venom decreased the expression levels of inflammation markers, including inducible nitric oxide synthase and cyclooxygenase-2. In addition, wasp venom decreased the nuclear translocation of nuclear factor κB (NF-κB), which is a key transcription factor in the regulation of cellular inflammatory response. Overall, the findings demonstrated that wasp venom inhibited LPS-induced inflammation in microglial cells by suppressing the NF-κB-mediated signaling pathway, which warrants further studies to confirm its therapeutic potential for neurodegenerative diseases.

**Abstract:**

The aim of this study was to compare the anti-inflammatory effect of wasp venom (WV) from the yellow-legged hornet (*Vespa velutina*) with that of bee venom (BV) on BV-2 murine microglial cells. WV was collected from the venom sac, freeze-dried, and used for *in vitro* examinations. WV and BV were non-toxic to BV-2 cells at concentrations of 160 and 12 µg/mL or lower, respectively. Treatment with WV reduced the secretion of nitric oxide and proinflammatory cytokines, including interleukin-6 and tumor necrosis factor alpha, from BV-2 cells activated by lipopolysaccharide (LPS). Western blot analysis revealed that WV and BV decreased the expression levels of inflammation markers, including inducible nitric oxide synthase and cyclooxygenase-2. In addition, WV decreased the nuclear translocation of nuclear factor κB (NF-κB), which is a key transcription factor in the regulation of cellular inflammatory response. Cumulatively, the results demonstrated that WV inhibited LPS-induced neuroinflammation in microglial cells by suppressing the NF-κB-mediated signaling pathway, which warrants further studies to confirm its therapeutic potential for neurodegenerative diseases.

## 1. Introduction

*Vespa velutina nigrithorax*, the invasive yellow-legged hornet, is indigenous to Southeast Asia [1]. It was first reported in France in the summer of 2004, presumably through accidental importation from China, although the actual route of its incursion into Europe remains unclear [2,3,4]. Since then, *V. velutina* has rapidly spread across Europe and Asia, and has colonized other countries worldwide [5,6,7]. Increases in wasp populations are concerning because of their potential impact on populations of beneficial, pollinating insects [3]. For instance, they have an intense predatory activity toward western honey bees (*Apis mellifera*), which have no effective defensive strategy against the predation pressure of *V. velutina* [5,8]. Thus, diverse strategies to control the population of *V. velutina* colonies are being considered [9,10,11,12]. In that context, this research explored the potential benefit that can be derived from abundant wasp populations by investigating the advantageous activities of wasp venom.

Hymenoptera venoms, including bee venom (BV) and wasp venom (WV), have attracted considerable interest owing to their therapeutic potential. Although the venoms are toxic to humans, the elucidation of their composition and working mechanisms has led to discoveries of their potential applications in treatment modalities for various disorders [13,14]. BV and WV have been widely studied, which has revealed significant concentrations of bioactive substances within their composition [13,15,16]. Among the venom components, melittin, apamin, and mastroparans have been well documented for their biological activities [14,17,18].

Various bioactive components have thus far been found in WV, although their composition and concentrations vary depending on the species of wasps and differ from those of BV [16,19]. The biologically active substances in WV are generally classified into three main groups: (i) high molecular weight proteins, including allergens and enzymes (such as hyaluronidase, α-glucosidase, and phospholipases); (ii) non-enzymatic small peptides, including mastoparans, wasp kinin, and antigen 5; and (iii) biogenic amines, including histamine, serotonin, and dopamine [13,16,19]. Certain components in WV are known to contribute to health-beneficial effects [20].

Multiple studies have demonstrated that similar to BV, WV can exert pain-relieving [21] and anti-arthritic activities [22]. Moreover, BV [23,24] and *V. tropica* venom [25] have been reported to suppress the inflammatory response in microglial cells. In particular, mast cell degranulating peptides (MCDPs), such as melittin and apamin in BV and mastoparans in WV, provide potent anti-inflammatory effects [14,26,27].

Investigation into the biological usefulness of *V. velutina* venom has revealed 293 putative toxin-encoding genes in the venom gland, of which neurotoxins represented the second-most abundant gene family [28]. Recently, the antioxidant activity of *V. velutina* venom has been examined in ultraviolet B-exposed HaCaT human keratinocytes [29]. In the present study, we investigated the anti-inflammatory potential of crude WV isolated from *V. velutina* in microglial cells through a comparison with the effect of BV.

Microglia, a type of glial cell, reside in the central nervous system (CNS) and play a phagocytic role in the innate immune system [30]. Microglial cells exquisitely respond to CNS injury and get activated along with undergoing morphological and phenotypical changes [31]. The persistent activation of microglial cells contributes to the neural damage and neurodegenerative disorders (such as Alzheimer’s disease, Parkinson’s disease, and amyotrophic lateral sclerosis), thus exacerbating pathological progression [31]. The activated microglial cells produce proinflammatory mediators and cytokines, such as nitric oxide (NO), inducible NO synthase (iNOS), cyclooxygenase-2 (COX-2), tumor necrosis factor α (TNFα), and interleukin-6 (IL-6) [32]. Numerous studies have demonstrated that inhibition of the inflammatory response in microglial cells provides therapeutic benefits in patients with neurodegenerative diseases [33,34]. Therefore, this study provides insights into the effectiveness of WV for the treatment and/or prevention of inflammation-associated neurodegenerative disorders.

## 2. Materials and Methods

### 2.1. Preparation of WV and BV

*V. velutina* colonies were collected in South Korea between August and October 2019, and were stored at −80 °C until use for the study. The venom was extracted by manually removing the venom sac from each wasp and subsequently eluting it through a Spin-X 0.45-μm cellulose acetate centrifuge tube filter (Corning Inc., Salt Lake City, UT, USA). The filtrate was then freeze-dried. A total of 124 mg of WV was obtained from approximately 650 adult female wasps (approximately 0.19 mg of venom per wasp). Next, 100 mg/mL lyophilized WV was dissolved in dimethyl sulfoxide for further experiments. BV powder was purchased from Chung Jin Biotech Co., Ltd. (Ansan, Korea).

### 2.2. Cell Culture

BV-2, an immortalized murine microglial cell line, originally developed by Dr. V. Bocchini at the University of Perugia (Perugia, Italy) [35,36], was generously provided by Dr. K. Suk at Kyungpook National University (Daegu, Korea). The cells were cultured in Dulbecco’s modified Eagle’s medium (Welgene, Gyeongsan, Gyeongbuk) containing 10% heat-inactivated fetal bovine serum (Welgene) and 1% penicillin-streptomycin (Welgene). The cell culture was maintained in a humidified incubator with 5% CO_2_ and 37 °C.

### 2.3. Cell Viability

For cell viability assay, cells were dispensed into 96-well plates at a density of 5 × 10^3^ cells/well. Next, the cells were treated with either BV (at 1, 2, 4, 8, 12, 16, and 20 µg/mL) or WV (at 10, 20, 40, 80, 120, 160, and 200 µg/mL) for 24 h. Finally, the number of live cells was measured using the Cell Counting Kit-8 (CCK-8; Dojindo Laboratories, Kumamoto, Japan), as previously described [37].

### 2.4. Determination of Nitric Oxide (NO) and Proinflammatory Cytokine Levels in Culture Medium

BV-2 cells were plated into a 6-well plate at a density of 5 × 10^5^ cells/well, activated with 10 ng/mL LPS, and treated with either WV or BV at the designated concentrations for 18 h. Prednisolone, an FDA-approved anti-inflammatory drug, was used as a positive control and treated at 10 µM. The culture media were collected and subjected to the quantification of NO using the Griess reagent (Promega, Madison, WI, USA) or the enzyme-linked immunosorbent assay (ELISA) for TNFα and IL-6 using commercially available kits (Mouse ELISA set for each cytokine, BD Biosciences, San Jose, CA, USA) [38]. The obtained values were normalized to the total amount of proteins.

### 2.5. Western Blot Analysis

BV-2 cells were plated in a 100-mm culture dish at a density of 2 × 10^6^ cells, activated with 10 ng/mL LPS, and treated with either WV or BV at the designated concentrations for 24 h. The cells were collected and fractionated using NE-PER^®^ Nuclear and Cytoplasmic Extraction Reagent Kits (Thermo Fisher Scientific, Rockford, IL, USA). The cytoplasmic and nuclear fractions of the proteins were blotted and analyzed for the expression levels of various proteins, as previously described [39,40]. The primary antibodies used were immunoglobulins against iNOS (Enzo Life Sciences, Farmingdale, NY, USA), COX-2 (Cell Signaling Technology, Danvers, MA, USA), NF-κB (BioWorld Technology, St. Louis, MN, USA), β-actin (Santa Cruz Biotechnology, Dallas, TX, USA), and Lamin B (Santa Cruz Biotechnology, Dallas, TX, USA). After allowing the appropriate secondary antibody (horseradish peroxidase conjugate) to interact with the primary antibody, protein bands were visualized using the SuperSignal*™* West Pico Chemiluminescent Substrate (Pierce, Cheshire, UK) and LAS4000 Mini (GE Healthcare Life Sciences, Little Chalfont, UK). The digitalized blot images were then densitometrically analyzed using Image-Studio Lite version 5.2 (LI-COR Biotechnology, Lincoln, NE, USA).

### 2.6. Luciferase Reporter Assay

To determine the transcriptional activity of the NF-kB response element (NRE) in microglial cells, BV-2 cells were transfected with the pGL4.32[luc2P/NF-κB-RE/Hygro] vector (Promega, Madison, WI, USA) using FuGENE^®^ HD Transfection Reagent (Promega). The transfectant carrying the NRE-luciferase reporter gene construct was named BV-2-NRE, and it was maintained in its growth medium containing 0.8 mM hygromycin (Sigma-Aldrich, St. Louis, MO, USA).

To perform the luciferase reporter assay, BV-2-NRE cells were plated into a 6-well plate at a density of 2 × 10^5^ cells/well and were treated with either WV at 40 µg/mL or BV at 4 µg/mL for 24 h. Next, 30 µM prednisolone was used as a positive control. Lipopolysaccharide (LPS; Sigma-Aldrich, St. Louis, MO, USA) was treated at 5 ng/mL for 6 h before the venom treatment was terminated. The cells were then collected and subjected to measurement of the NRE-luciferase activity using a luciferase assay system (Promega, Madison, WI, USA) [41].

### 2.7. Statistical Analysis

Statistical analyses were performed using SPSS software version 23.0 (SPSS Inc., Chicago, IL, USA). Statistical differences among the means were tested by Student’s *t*-test. *p* values of less than 0.05 were considered significant. Statistically significant differences between the values were indicated by asterisks (*) and hashtags (#).

## 3. Results and Discussion

**Figure 1:**
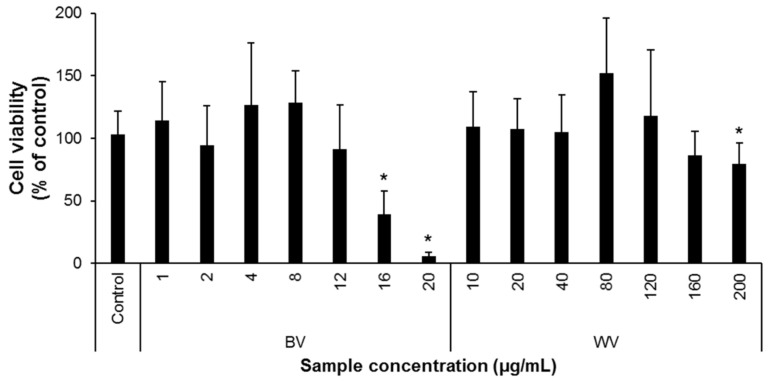
Cytotoxicity of bee venom (BV) and wasp venom (WV) in BV-2 microglial cells. BV-2 cells were plated into a 96-well plate at a density of 5 × 10^3^ cells/well and were treated with either BV or WV at the designated concentrations for 24 h. The cell viability was then assayed using the CCK-8 kit. BV and WV were non-toxic at concentrations of ≤12 µg/mL and ≤160 µg/mL, respectively. Independent experimental sessions (*N*) = 5; error bars, mean ± standard deviation (SD). Asterisks (*) indicate a statistically significant difference compared to the control (*p* < 0.05; Student’s *t*-test).

**Figure 2:**
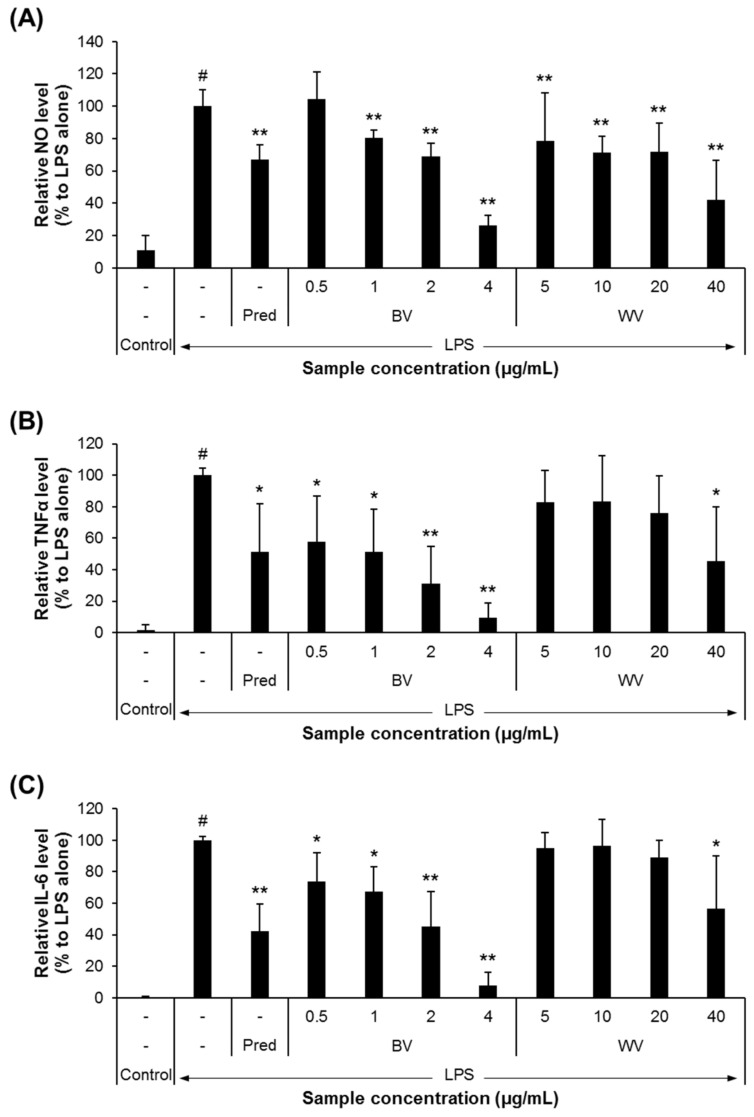
WV decreased the production of nitric oxide (NO) and secretions of proinflammatory cytokines from lipopolysaccharide (LPS)-treated BV-2 cells. (**A**‒**C**) BV-2 cells were plated into a 6-well plate at a density of 5 × 10^5^ cells/well, activated with 10 ng/mL of LPS, and treated with either WV or BV at the designated concentrations for 18 h. Prednisolone (Pred, 10 µM) was used as the positive control. The culture media were collected and subjected to the quantification of NO (**A**), tumor necrosis factor *α* (TNFα) (**B**), and interleukin-6 (IL-6) (**C**) levels. Treatment of LPS-stimulated BV-2 cells with either WV at ≥5 µg/mL or BV at ≥1 µg/mL significantly decreased NO production. In addition, WV at >20 µg/mL or BV at ≥0.5 µg/mL reduced the levels of proinflammatory cytokines TNFα and IL-6 secreted from LPS-stimulated BV-2 cells. *N* = 5; error bars, mean ± SD. Statistically significant differences between the means were analyzed by Student’s *t*-test. Hashtags (^#^) indicate a significant difference compared to the control (*p* < 0.05). Asterisks (*) indicate a significant difference compared to the LPS alone treatment (*, *p* < 0.05; **, *p* < 0.01).

**Figure 3:**
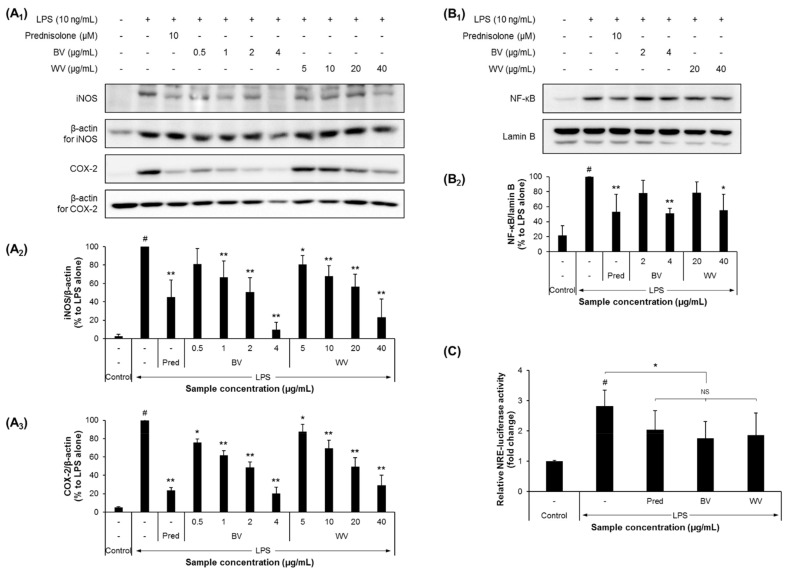
WV decreased the expression of inflammation-related proteins and decreased NF-kB response element (NRE) transcription activity in BV-2 cells. (**A**,**B**) BV-2 cells were plated in a 100-mm culture dish at a density of 2 × 10^6^ cells, activated with 10 ng/mL of LPS, and treated with either WV or BV at the designated concentrations for 24 h. Prednisolone (Pred, 10 µM) was used as the positive control. The cells were collected, lysed, and used to measure the relative expression levels of cytoplasmic iNOS and COX-2 (**A**) and nuclear NF-κB (**B**). (**C**) BV-2-NRE cells carrying the NRE sequence linked to the luciferase reporter gene were plated into a 6-well plate at a density of 2 × 10^5^ cells/well and were treated with either WV at 40 µg/mL or BV at 4 µg/mL for 24 h. Prednisolone (30 µM) was used as the positive control. LPS was treated at 5 ng/mL for 6 h before the venom treatment was terminated. The cells were then collected and subjected to the measurement of the NRE-luciferase activity. *N* = 4 for (**A**,**B**) and 3 for (**C**); error bars, mean ± SD. Statistically significant differences between the means were analyzed by Student’s *t*-test. Hashtags (^#^) indicate a significant difference compared to the control (*p* < 0.05). Asterisks (*) indicate a significant difference compared to the LPS alone treatment (*, *p* < 0.05; **, *p* < 0.01). NS, not significant.

The biologically advantageous effects of BV of honey bee, such as anti-neuroinflammatory, neuroprotective, and cognition-improving effects, as well as its active components have been well documented by extensive studies over the past decades [23,42,43,44,45]. In the present study, compared with BV, the anti-inflammatory effect of WV obtained from *V. velutina* was examined in BV-2 murine microglial cells. We found that WV was at least 13-fold less cytotoxic than BV (Figure 1). In addition, WV significantly decreased the secretions of NO, TNFα, and IL-6 at concentrations of >20 µg/mL, whereas BV was effective at concentrations of ≥1 µg/mL in BV-2 cells (Figure 2). WV also inhibited the expression of iNOS and COX-2 at concentrations of ≥10 µg/mL, whereas BV did at concentrations of ≥0.5 µg/mL by suppressing the transcriptional activity of NF-κB in LPS-stimulated BV-2 cells (Figure 3). WV was approximately 20-fold weaker than BV in most inflammatory parameters, which suggests that the component(s) responsible for anti-neuroinflammation exist at significantly lower concentrations in WV than in BV. Identification of the active substances in WV and their specific working mechanisms await further study.

Neuroinflammation is closely related to neurodegenerative diseases [34,46]. Acute inflammatory response is an innate host defense mechanism that protects the brain from pathogens and toxins, promotes neural tissue repair, and clears death cell debris [47,48]. However, it is commonly appreciated that sustained neuroinflammation is triggered with the activation of microglia at the site of brain injury or neural degeneration [49,50]. The activated microglial cells secrete various inflammatory factors, including cytokines and complement components, which together induces activation of astrocytes. The reactive astrocytes are involved in neuronal death in those with neurological conditions and in aged brain [34,51]. Thus, microglia-mediated inflammation is considered a critical neurotoxic event in the pathogenesis of neurodegenerative disorders. Numerous studies have demonstrated that the blocking of the activation of microglial cells and subsequently reduced production of proinflammatory factors attenuated neuronal cell loss [52,53,54,55].

The BV-2 cell line is most commonly used to study neuroinflammation *in vitro* because the cells are genetically, phenotypically, and cytophysiologically similar to primary microglia [56]. For instance, BV-2 cells express the ionized calcium-binding adaptor protein-1 (a microglia activation marker), but not galactocerebroside (an oligodendrocyte marker) or glial fibrillary acidic protein (an astrocyte marker), and produce a substantial amount of NO and proinflammatory cytokines, including TNFα, IL-6, and IL-1β when stimulated with LPS [57,58]. Consistent with these previous reports, our result demonstrated that the secretion levels of NO, TNFα, and IL-6 from BV-2 cell culture were significantly increased by LPS treatment. Moreover, the elevated levels were significantly decreased by prednisolone (a positive control) and concentration-dependently by WV or BV. However, in our culture setting, the secretory IL-1β level was not clearly detectable, although LPS-treated BV-2 cells express IL-1β mRNA according to the literature [35,59]. The extent of IL-1β secretion from BV-2 cells appears to vary depending on different contexts [60,61].

Prednisolone is the most frequently used glucocorticoid owing to its anti-inflammatory and immunosuppressive activities [62,63,64]. Considering the anti-inflammatory mechanisms of prednisolone [65,66,67,68] and BV [23,42,69], it was assumed that the suppressive activities of WV on NO production and cytokine release would be mediated through the NF-κB pathway. Indeed, our results from western blot analysis and luciferase reporter assay exhibited significantly increased protein levels and transcriptional activity of nuclear NF-κB and the cytoplasmic levels of its downstream effectors iNOS and COX-2 in LPS-treated BV-2 cells, whereas WV decreased the levels in a concentration-dependent manner.

LPS can strongly stimulate microglial activation and thus ignite inflammatory responses that promote disease progression in various models of neurodegeneration [70,71,72]. LPS interacts with toll-like receptors such as TLR4 on the surface of microglia. The activated TLR4 signaling then modulates various transcription factors, including NF-κB which triggers the production and release of proinflammatory cytokines [73,74,75]. In this sense, WV was presumed to interfere with the TLR4/NF-κB signaling pathway in microglial cells, thereby preventing neuroinflammation-related neural damage.

Regarding the bioactive components in WV, certain types of MCDPs are promising candidates because dried WV is composed of approximately 70% peptides [18]. A couple of review articles addressed the possible therapeutic potential of mastoparans, which are generally found in vespid venoms, for the treatment of neurodegenerative disorders [14,17]. Considering that the main venom peptides and proteins vary depending on the species and sociality of wasps [16], it cannot be excluded that there may be unknown substances that would be effective in suppressing microglial activation. Thus, the identification of active compounds in WV and the elucidation of their working mechanism(s) await further study.

In conclusion, the findings of this study demonstrated that WV from *V. velutina* inhibited LPS-induced inflammation in BV-2 microglial cells by suppressing the NF-κB-mediated signaling pathway. This provides insights into the usefulness of WV for preventive and ameliorative applications in neurodegenerative diseases such as Parkinson’s and Alzheimer’s diseases.

## Data Availability

The authors declare that all data supporting the findings of this study are available in the article and can be provided by the corresponding author on reasonable request.
